# Comparison of intraepidermal nerve fiber density and confocal corneal microscopy for neuropathy

**DOI:** 10.1002/acn3.52218

**Published:** 2024-10-12

**Authors:** Evan L. Reynolds, Fallon Koenig, Maya Watanabe, Alyssa Kwiatek, Melissa A. Elafros, Amro Stino, Don Henderson, David N. Herrmann, Eva L. Feldman, Brian C. Callaghan

**Affiliations:** ^1^ Department of Epidemiology and Biostatistics Michigan State University East Lansing Michigan 48823 USA; ^2^ Department of Neurology University of Michigan Ann Arbor Michigan 48104 USA; ^3^ Department of Biostatistics University of Michigan Ann Arbor Michigan 48104 USA; ^4^ Department of Neurology University of Rochester Rochester New York 14642 USA

## Abstract

**Objective:**

Compare the diagnostic characteristics of intraepidermal nerve fiber density (IENFD) and confocal corneal microscopy (CCM) for distal symmetric polyneuropathy (DSP) and small fiber neuropathy (SFN).

**Methods:**

Participants with obesity were recruited from bariatric surgery clinics and testing was performed prior to surgery. DSP and SFN were determined using the Toronto consensus definitions of probable neuropathy. IENFD was assessed from 3 mm punch biopsies of the distal leg and proximal thigh. CCM was performed on both eyes with manual and automated counting. The Michigan Neuropathy Screening Instrument questionnaire (MNSIq) was also completed. Diagnostic capability was determined using areas under the receiver operating characteristics curve (AUC) from logistic regression.

**Results:**

We enrolled 140 participants (mean [standard deviation [SD]] age: 50.3 years [7.1], 77.1% female, BMI: 44.4 kg/m^2^ [6.7]). In this population, 22.9% had DSP and 14.3% had SFN. Distal leg IENFD had the largest AUC (95% confidence interval) for DSP (0.78, 0.68–0.89) and SFN (0.85, 0.75–0.96). Proximal thigh IENFD (DSP: AUC: 0.59, 0.48–0.69, SFN: AUC: 0.59, 0.46–0.73) and CCM metrics (DSP: AUC range: 0.55–0.60, SFN: AUC range: 0.45–0.62) had poorer diagnostic capability than distal leg IENFD for DSP/SFN (*P* < 0.05). MNSIq had similar diagnostic capability to distal leg IENFD for both DSP/SFN (DSP: AUC: 0.76, 0.68–0.85, SFN: AUC: 0.81, 0.73–0.88). More participants (52%) preferred skin biopsies to CCM.

**Interpretation:**

Distal leg IENFD was the best quantitative measure of DSP/SFN. CCM had poor diagnostic characteristics and fewer patients preferred this test to IENFD. The MNSIq had similar diagnostic characteristics to distal leg IENFD, indicating its value as a diagnostic tool in the clinical setting.

**Clinical Trial Registration:**

clinicaltrials.gov: NCT03617185.

## Introduction

Distal symmetric polyneuropathy (DSP), a symmetric, length‐dependent damage to peripheral nerves, is a highly prevalent condition that lowers quality‐of‐life and increases mortality.^1^, ^2^ Small fiber neuropathy (SFN), damage specifically to small unmyelinated fibers, is considered an early pattern of peripheral nerve injury in the development of DSP and a hallmark of neuropathic pain.^3^ The gold standard quantitative measurement for SFN is the assessment of intraepidermal nerve fiber density (IENFD) from skin punch biopsies^4^, ^5^, ^6^ and is well established in clinical research studies.^4^, ^7^, ^8^, ^9^ However, recently, confocal corneal microscopy (CCM) has emerged as a less invasive alternative to skin biopsies with potential to detect SFN.^10^ A meta‐analysis of 38 studies comprising approximately 4000 participants found multiple CCM parameters were significantly reduced in individuals with versus without neuropathy.^11^ Consequently, use of CCM as a diagnostic tool for neuropathy in research studies has increased substantially over the past decade. From 2013 to 2023, the number of PubMed‐indexed scientific articles that included the search terms “confocal corneal microscopy” and “neuropathy” increased from 16 in 2013 to 48 in 2023, representing a 200% rise. During that same period, articles that included “intraepidermal nerve fiber density” and “neuropathy” only increased by 29%, from 34 articles in 2013 to 44 in 2023.

Despite the more frequent use of CCM as a research diagnostic tool for neuropathy, its comparative performance to IENFD has not been firmly established. Two studies (*n* = 88 and *n* = 89) found CCM had superior diagnostic capability for neuropathy in persons with Type 1 diabetes compared to IENFD, with areas under the receiver operating characteristic (ROC) curves (AUC) ranging from 0.77 to 0.82 for CCM parameters and 0.66 to 0.73 for IENFD.^12^, ^13^ In contrast, three studies (*n* = 214, *n* = 680, and *n* = 168) found that CCM parameters had poorer diagnostic characteristics for neuropathy versus IENFD in participants with Type 2 diabetes (AUC ranges: CCM: 0.52 to 0.68, IENFD: 0.71 to 0.74).^14^, ^15^, ^16^ Given this conflicting evidence, more studies comparing the diagnostic characteristics of IENFD versus CCM are needed in order to recommend the best diagnostic tool for neuropathy in both research and clinical practice. Importantly, the diagnostic capabilities of these assessments have not been directly compared in a population with obesity, which is a critical knowledge gap given that obesity is the second most important metabolic risk factor for neuropathy after diabetes.^17^ Additionally, patient preferences between IENFD and CCM remain unknown, which limits applicability to the clinical setting.

In the present study, we compared the diagnostic utility of IENFD and CCM, for DSP and SFN, using baseline data from a cohort of persons with obesity enrolled in a clinical trial of bariatric surgery and exercise at the University of Michigan. We also evaluated the diagnostic utility of the Michigan Neuropathy Screening Instrument questionnaire (MNSIq) to compare this simple questionnaire to these more invasive tests, IENFD and CCM. In addition, among persons with DSP and SFN, we assessed correlations between IENFD, CCM, and other clinical neuropathy scales. Finally, we ascertained patient preferences between skin biopsies and CCM.

## Methods

### Population and study design

From October 2018 to July 2022, 140 participants were enrolled from a clinical trial “Effect of Exercise and Surgical Weight Loss on Polyneuropathy” (clinicaltrials.gov: NCT03617185). Specifically, participants were recruited from three bariatric surgery clinics in Southeast Michigan, United States (US), including Michigan Medicine, Henry Ford Health System, and Trinity Health. Study inclusion criteria were attendance at a bariatric surgery clinic, age ≥40 years, and BMI > 35 kg/m^2^ with one comorbid condition or BMI > 40 kg/m^2^ without a comorbid condition. Comorbid conditions included history of organ transplant, chronic kidney disease, tuberculosis, history of abnormal cardiovascular stress test, congestive heart failure class III, rheumatoid arthritis, human immunodeficiency virus, Sjogren's, cancer/chemotherapy, hepatitis B/C, alcoholism, lupus, sarcoid, thyroid‐stimulating hormone, autoimmune disorders, vitamin B12 deficiency, vitamin B1 deficiency, corneal transplant, cognitive impairment, and others on a case by case basis. Study exclusion criteria were use of anticoagulants, exercise stress test failure, use of a walking assistance device, current smoking, weight >450 lb, and other factors that have been previously described.^18^ The present cross‐sectional observational study includes baseline data from enrolled clinical trial participants prior to starting study interventions (bariatric surgery and/or high‐intensity interval training).

### Outcomes

DSP and SFN were determined according to the Toronto consensus definitions of probable neuropathy, as determined by one of the nine neuromuscular specialists at the University of Michigan.^19^ We included SFN as a separate outcome from DSP given that IENFD and CCM are proposed as assessments of small nerve fiber function. According to the Toronto definition, a diagnosis of DSP required the presence of at least two of the following: neuropathy symptoms, abnormal sensory examination (decreased pinprick and/or decreased vibration sensation), and abnormal reflexes.^19^ Similarly, a diagnosis of probable small fiber involved neuropathy (SFN) required presence of neuropathy symptoms and an abnormal sensory examination (any degree of decreased pinprick sensation). We did not require participants to have isolated SFN (e.g., absence of large fiber neuropathy signs). We also determined whether participants met the Toronto consensus definition for definite DSP, which required abnormal nerve conduction study (NCS) parameters on at least two separate nerves (tibial, peroneal, and sural).^20^ Abnormal NCS parameters were determined using the 3rd/97th percentile among published normative data.^21^

### Intraepidermal nerve fiber density

IENFD (unit = fibers/mm) was assessed at the distal leg (10 cm proximal to the lateral malleolus) and proximal thigh (20 cm distal to the anterior superior iliac spine) via a 3‐mm skin punch biopsy. Prior to the skin biopsy, participants were given a lidocaine injection at each biopsy site. Skin biopsies were fixed in Zamboni's fixative for 12–24 h and then cryoprotected in phosphate buffered 20% glycerol prior to shipping to University of Rochester for processing. The skin biopsies were sectioned at 50 μm thickness using a sliding freezing microtome and four sections/biopsy (selected using systematic random sampling) were stained with monoclonal antibodies to human PGP 9.5 (BIO‐RAD, Hercules, CA, USA) according to previously described brightfield techniques.^7^, ^8^, ^9^ Individual nerve fibers that crossed into the epidermis in each of the four sections were counted by a blinded examiner using an established protocol, summed, and the divided by the length of the epidermis to determine the IENFD (fibers/mm) for each biopsy.^4^ We also calculated the IENFD of the distal leg to IENFD of the proximal thigh ratio.

### Confocal corneal microscopy

CCM was completed on both eyes using HRT3RCM by Heidelberg Engineering. The operator applied two to three numbing drops and a gel lubricant in each eye. Once images were captured, the operator selected the four clearest images for each eye according to an established protocol with high inter‐rater reliability.^22^ High clarity images were selected using the criteria of depth, focus position, and contrast. Further, to ensure consistency of image selection across all subjects, one image was selected from each of three different regions of the cornea (medial, lateral, and superior). When multiple images meeting that criteria were available, the one with the greatest number of corneal nerves was selected. The images were averaged, garnering a more global representation of the corneal nerves. Images were analyzed using the software programs CCMetrics and ACCMetrics. Using CCMetrics, the operator manually traces the corneal nerve fibers and branches (manual), whereas in ACCMetrics the software performs the tracing (automated). Values from the four images were averaged to obtain the values for the respective eye.

Manual and automated CCM parameters included the corneal nerve fiber density (CNFD, unit = fibers/mm^2^), corneal nerve branch density (CNBD, unit = branches/mm^2^), and corneal nerve fiber length (CNFL, unit = mm/mm^2^), and manual CCM additionally measured tortuosity coefficient (TC), a measure of nerve fiber distortion in the cornea.^23^ Automatic CCM analysis as previously described^24^ reassessed the above parameters to also calculate corneal total branch density (CTBD, unit = no./mm^2^), corneal nerve fiber area (CNFA, unit = mm/mm^2^), and corneal nerve fiber width (CNFW, unit = mm/mm^2^), as well as corneal nerve fractal dimension (CNFrD, unit = number of dimensions), a measure of nerve fiber topological complexity in the cornea.^25^ For each participant, CCM parameters were averaged between the left and right measurements. If participants were missing a left or right CCM measurement, the non‐missing value was used in place of the average.

### DSP questionnaire

The MNSIq, a questionnaire consisting of 15 questions to assess for DSP, was administered.^26^

### Clinical DSP scales

The Utah Early Neuropathy Scale (UENS) total score and the total modified Toronto Clinical Neuropathy Score (mTCNS) sensory score were assessed as previously described.^27^, ^28^

### Clinical SFN scales

For SFN, the total score from the pin sensation section of the UENS scale (range: 0–24) and the total score on the pinprick and temperature sections of the sensory examination of the mTCNS (range: 0–6) were assessed.

### Participant preference: Skin biopsy versus CCM

Skin biopsy and CCM were completed in no particular order; after both were completed, participants were asked by the research coordinator which test they preferred.

### Statistical analysis

Descriptive statistics were used to summarize participants' demographic information. The primary analysis determined the discriminatory capability of IENFD and CCM metrics for DSP and SFN. Specifically, a series of univariate logistic regression models were fit separately for DSP and SFN as a function of (1) IENFD of the distal leg, (2) IENFD of the proximal thigh, (3) IENFD distal leg to IENFD proximal thigh ratio, (4) CNFD (manual and automated), (5) CNBD (manual and automated), (6) CNFL (manual and automated), (7) TC (manual), (8) CTBD (automated), (9) CNFrD (automated), (10) CNFA (automated), (11) CNFW (automated), and (12) MNSIq. To assess the discriminatory capability of the different assessments, ROC curves were constructed for each model and summarized by calculating AUC. DeLong's method was used to determine 95% confidence intervals (CI) of the resulting AUC. Delong's paired test for correlated ROC curves was used to determine differences of resulting AUCs between different IENFD and CCM metrics, for DSP and SFN, separately. To determine whether IENFD and CCM parameters have different diagnostic characteristics for definite DSP, as a sensitivity analysis, we re‐fit our primary univariate logistic regression models for definite DSP, and re‐calculated AUC.

Pearson's correlation coefficients were also calculated to assess correlation among DSP and SFN measures. Specifically, among participants with DSP, correlations were determined between IENFD parameters, CCM parameters, UENS, and the total mTCNS sensory score. Among participants with SFN, correlations were determined between IENFD parameters, CCM parameters, UENS pinprick score, and mTCNS pinprick and temperature score.

Available case analysis handled missing data. All analyses were completed using R software version 4.2.1.

### Standard protocol approvals, registrations, and participant consents

This study was approved by the University of Michigan Medical School Institutional Review Board. All study participants provided written informed consent.

## Results

### Study participation, demographic information, and missing data

There were 140 participants enrolled in this study. The mean (SD) age was 50.3 (7.1) years and 77.1% were female (Table 1). Participant race was primarily White (69.3%) or Black (24.3%). Three (2.1%) participants reported ethnicity as Hispanic/Latino. The mean (SD) BMI was 44.4 (6.7) kg/m^2^, 44 (31.4%) had Type 2 diabetes, and 61 (43.6%) had pre‐diabetes. We found that 32 (22.9%) of participants had probable DSP, 20 (14.3%) had definite DSP, and 20 (14.3%) had SFN. Several study participants had sporadic missing data. Specifically, 7 participants were missing CCM assessments, 4 were missing IENFD of the distal leg, 3 were missing IENFD of the proximal thigh, 5 were missing the IENFD distal leg to IENFD proximal thigh ratio, 2 was missing MNSIq, 2 were missing UENS, and 2 were missing mTCNS.

**Table 1:** Demographic and socioeconomic characteristics of study participants.

Variable	All participants (*N* = 140)	Distal symmetric polyneuropathy (*N* = 32)	Small fiber neuropathy (*N* = 20)
Age, mean (SD) (years)	50.3 (7.1)	54.1 (6.7)	53.2 (6.8)
Sex, *N* (%) female	108 (77.1)	18 (56.3)	9 (45.0)
Race, *N* (%)			
White	97 (69.3)	25 (95.0)	19 (95.0)
Black	34 (24.3)	5 (15.6)	1 (5.0)
Asian	2 (1.4)	1 (3.1)	0 (0.0)
Native American	1 (0.7)	0 (0.0)	0 (0.0)
Multi‐racial/other	5 (3.5)	1 (3.1)	0 (0.0)
Unknown	1 (0.7)	0 (0.0)	0 (0.0)
Ethnicity, *N* (%) Hispanic/Latino	3 (2.1)	1 (3.1)	0 (0.0)
Smoking status, *N* (%)			
Current smoker	2 (1.4)	0 (0.0)	0 (0.0)
Ex‐smoker	51 (36.4)	13 (40.6)	6 (30.0)
Never smoker	84 (60.0)	19 (59.4)	14 (70.0)
Unknown	3 (2.1)	0 (0.0)	0 (0.0)
Marital status, *N* (%)			
Married	73 (52.1)	23 (71.9)	13 (50.0)
Single	35 (25.0)	7 (21.9)	5 (25.0)
Divorced	21 (15.0)	1 (3.1)	1 (5.0)
Widowed	5 (3.6)	1 (3.1)	1 (5.0)
Significant other	4 (2.9)	0 (0.0)	0 (0.0)
Education, *N* (%)			
Professional or graduate degree	28 (20.0)	4 (12.5)	3 (15.0)
College degree	57 (40.7)	15 (46.9)	7 (35.0)
Some college or vocational college	41 (29.3)	10 (31.3)	7 (35.0)
High school graduate, GED or less	11 (7.8)	3 (9.4)	3 (15.0)
Employment status, *N* (%)			
Employed	109 (77.9)	24 (75.0)	16 (80.0)
Retired	11 (7.9)	7 (21.9)	3 (15.0)
Seeking work	3 (2.1)	1 (3.1)	1 (5.0)
Keeping house	7 (5.0)	0 (0.0)	0 (0.0)
Student	1 (0.7)	0 (0.0)	0 (0.0)
Other	6 (4.3)	0 (0.0)	0 (0.0)
Insurance, *N* (%)			
Private insurance	111 (79.3)	29 (90.6)	17 (85.0)
Medicare/medicaid	19 (13.6)	0 (0.0)	1 (5.0)
Other	7 (5.0)	3 (9.4)	2 (10.0)
Unknown	3 (2.1)	0 (0.0)	0 (0.0)
Body mass index, mean (SD)	44.4 (6.7)	43.6 (6.4)	44.6 (6.1)
Diabetes status, *N* (%)			
Diabetes	44 (31.4)	18 (56.2)	14 (70.0)
Pre‐diabetes	61 (43.6)	9 (43.6)	4 (20.0)

### Diagnostic characteristics for DSP

**Figure 1:**
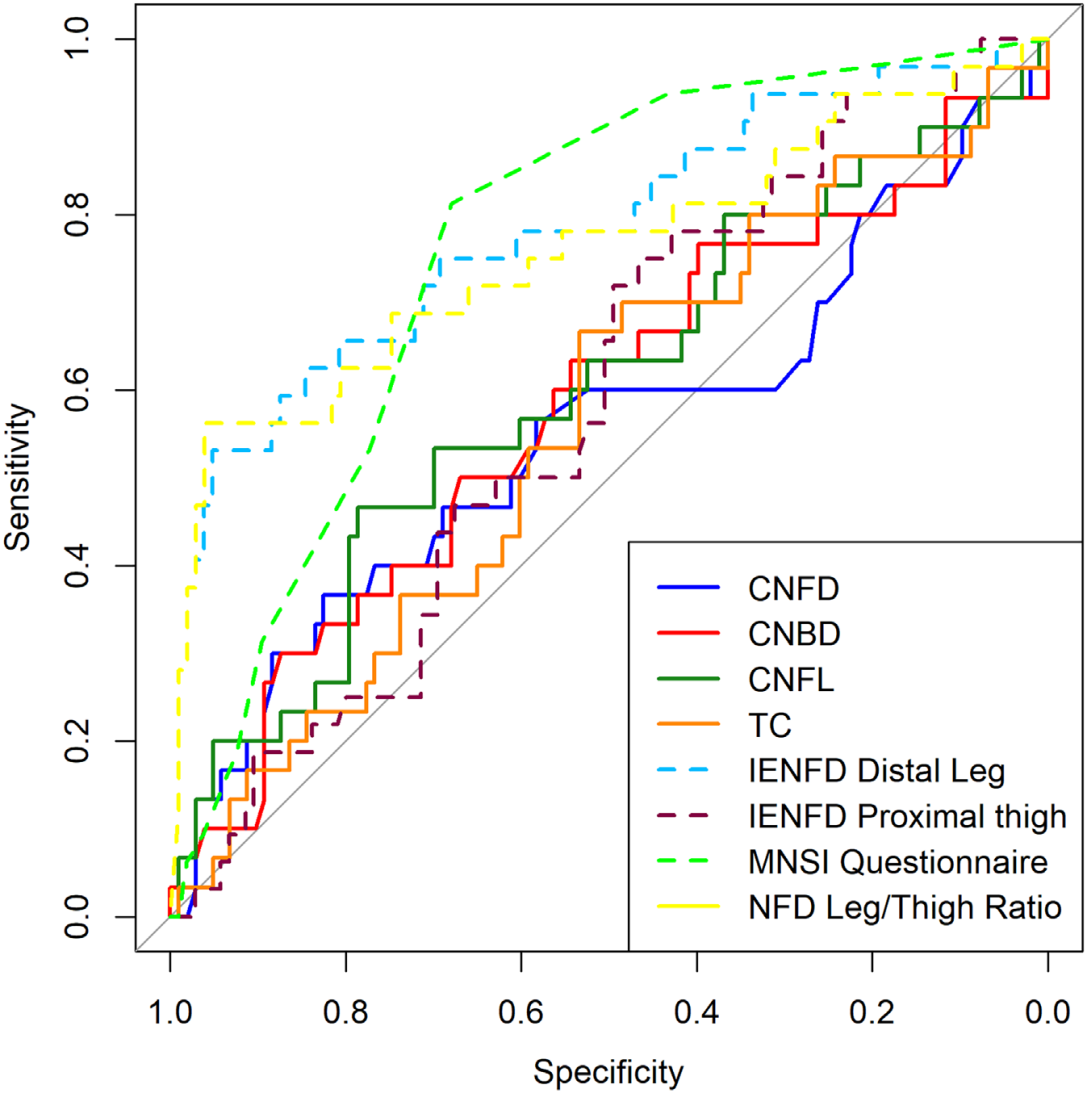
Receiver operator characteristic curves for IENFD, manual CCM, and MNSI Questionnaire diagnostic tests of DSP. CNBD, corneal nerve branch density; CNFD, corneal nerve fiber density; CNFL, corneal nerve fiber length; IENFD, intraepidermal nerve fiber density; MNSI, Michigan Neuropathy Screening Instrument; TC, tortuosity coefficient.

IENFD of the distal leg had the highest diagnostic capability for DSP with the highest AUC (AUC: 0.78, 95% CI: 0.68–0.89; Table 2, Fig. 1). IENFD of the distal leg had similar diagnostic capability to the IENFD leg/thigh ratio (AUC: 0.76, 95% CI: 0.65–0.87) (*P* = 0.75), but outperformed IENFD of the proximal thigh (AUC: 0.59, 95% CI: 0.48–0.69) (*P* < 0.05) as well as all CCM parameters, which had similar diagnostic capabilities among them, with AUCs ranging from 0.55 (95% CI: 0.42–0.68) for manual CNFD to 0.60 (95% CI: 0.48–0.72) for manual/automated CNFL. The MNSIq (AUC: 0.76, 95% CI: 0.68–0.85) had the third largest AUC for DSP after IENFD of the distal leg which did not differ significantly (*P* = 0.78).

**Table 2:** Diagnostic characteristics of IENFD and CCM measurements for DSP and SFN.

Variable	Distal symmetric polyneuropathy AUC (95% CI)	Small fiber neuropathy AUC (95% CI)
IENFD metrics		
IENFD distal leg	0.78 (0.68–0.89)	0.85 (0.75–0.96)
IENFD proximal thigh	0.59 (0.48–0.69)	0.59 (0.46–0.73)
IENFD distal leg to proximal thigh ratio	0.76 (0.65–0.87)	0.82 (0.68–0.96)
Manual CCM		
CNFD	0.55 (0.42–0.68)	0.55 (0.40–0.70)
CNBD	0.58 (0.46–0.71)	0.53 (0.39–0.67)
CNFL	0.60 (0.48–0.72)	0.59 (0.45–0.73)
TC	0.56 (0.44–0.68)	0.62 (0.48–0.76)
Automated CCM		
CNFD	0.57 (0.44,0.69)	0.53 (0.39,0.68)
CNBD	0.56 (0.43,0.68)	0.50 (0.36,0.65)
CNFL	0.60 (0.48,0.72)	0.58 (0.43,0.72)
CTBD	0.58 (0.45,0.70)	0.53 (0.39,0.68)
CNFrD	0.58 (0.45,0.70)	0.45 (0.31,0.59)
CNFA	0.58 (0.46,0.70)	0.62 (0.48,0.75)
CNFW	0.57 (0.44,0.69)	0.57 (0.42,0.72)
Other neuropathy scales		
MNSI questionnaire	0.76 (0.68–0.85)	0.81 (0.73–0.88)

AUC, area under the receiver operating characteristics curve; CNBD, corneal nerve branch density; CNFA, corneal nerve fiber area; CNFD, corneal nerve fiber density; CNFL, corneal nerve fiber length; CNFrD, corneal nerve fractal dimension; CNFW, corneal nerve fiber width; CTBD, corneal total branch density; IENFD, intraepidermal nerve fiber density; MNSI, Michigan Neuropathy Screening Instrument; TC, tortuosity coefficient.

Sensitivity analyses revealed that diagnostic characteristics of IENFD of the proximal thigh and CCM parameters were similar for probable DSP and definite DSP. However, the diagnostic characteristics for IENFD of the distal leg and had improved diagnostic characteristics for definite DSP (AUC: 0.86, 95% CI: 0.75–0.97) compared to probable DSP (AUC: 0.78, 95% CI: 0.68–0.89).

### Diagnostic characteristics for SFN

**Figure 2:**
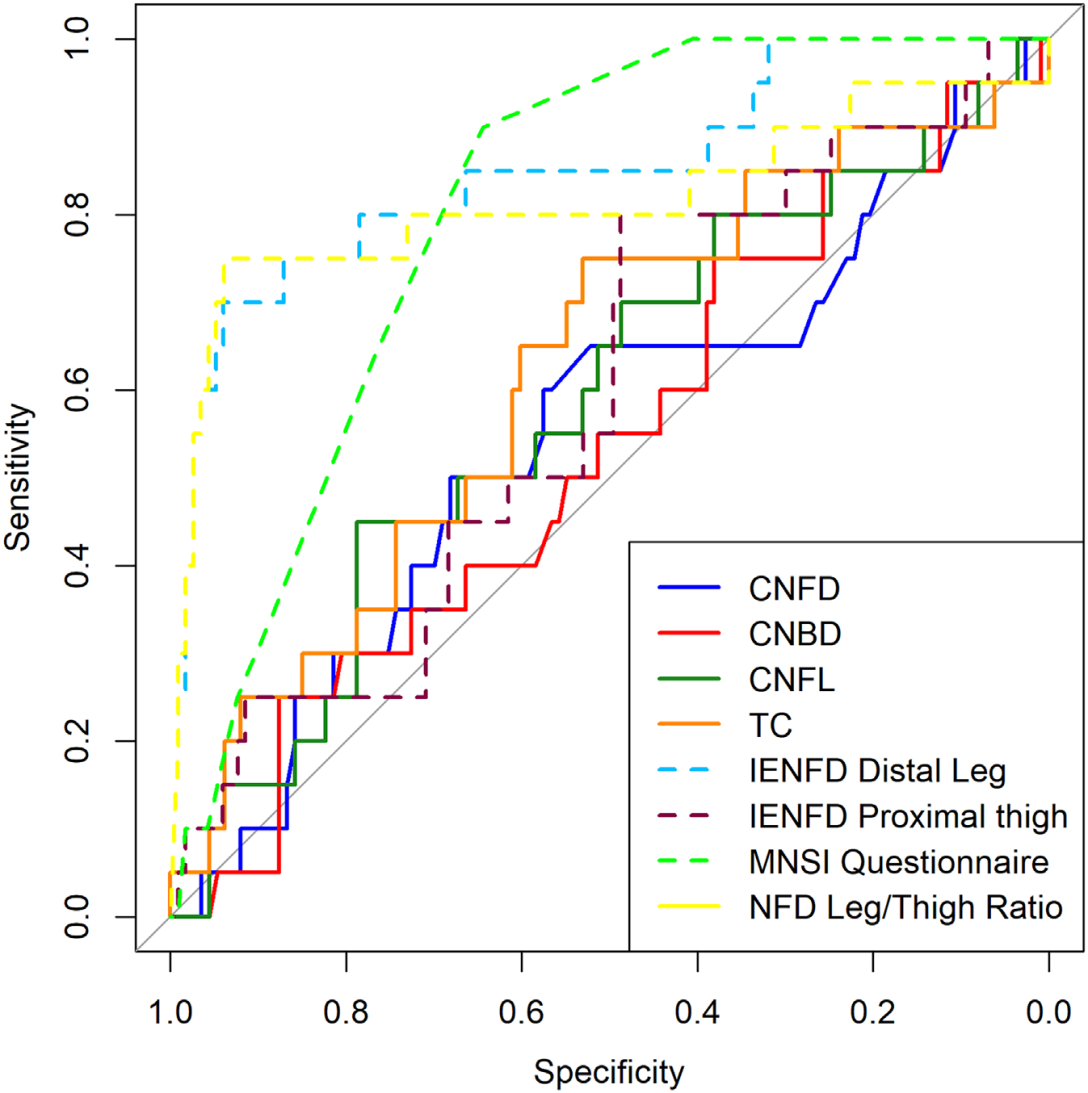
Receiver operator characteristic curves for IENFD, manual CCM, and MNSI Questionnaire diagnostic tests of SFN. CNBD, corneal nerve branch density; CNFD, corneal nerve fiber density; CNFL, corneal nerve fiber length; IENFD, intraepidermal nerve fiber density; MNSI, Michigan Neuropathy Screening Instrument; TC, tortuosity coefficient.

IENFD of the distal leg had the best diagnostic characteristics for SFN (AUC: 0.85, 95% CI: 0.75–0.96; Table 2, Fig. 2), which was also higher than the AUC of IENFD of the distal leg for DSP, as might be anticipated for a metric that assesses small fiber density. IENFD of the distal leg had similar diagnostic capability to the IENFD leg/thigh ratio (AUC: 0.82, 95% CI: 0.68–0.96) (*P* = 0.69). Notably, the AUC for IENFD of the distal leg was significantly larger (*P* < 0.05) than the AUC for IENFD of the proximal thigh as well as all CCM parameters, which had similar diagnostic capabilities among them, with AUCs ranging from 0.45 (95% CI: 0.31–0.59) for automated CNFrD to 0.62 (95% CI: 0.48–0.76) for manual TC and 0.62 (95% CI: 0.48–0.75) for automated CNFA. IENFD of the proximal thigh had similar diagnostic capability (AUC: 0.59, 95% CI: 0.46–0.73) to CCM parameters. The MNSIq (AUC: 0.81, 95% CI: 0.73–0.88) had the third largest AUC for SFN, which did not differ significantly from the AUC for IENFD of the distal leg (*P* = 0.46).

### Correlation among DSP measurements

Among individuals with DSP (*n* = 32), there were significant positive correlations (*r*) between manual CCM parameters (CNFD vs. CNBD: *r* = 0.74, CNFD vs. CNFL: *r* = 0.85, CNBD vs. CNFL: *r* = 0.91; Table 3). Manual CCM parameters did not correlate with IENFD or other clinical DSP scales (all *P* > 0.05). IENFD of the distal leg significantly and negatively correlated with UENS total score (*r* = −0.52) and mTCNS sensory score (*r* = −0.49), but not with IENFD of the proximal thigh or manual CCM metrics. Similarly, IENFD leg/thigh ratio significantly and negatively correlated with UENS total score (*r* = −0.37) and mTCNS sensory score (*r* = −0.39).

**Table 3:** Correlation among DSP/SFN measures for participants with DSP (*n* = 32) and SFN (*n* = 20).

DSP (*n* = 32)	IENFD distal leg	IENFD proximal thigh	IENFD distal leg to proximal thigh ratio	CNFD (manual)	CNBD (manual)	CNFL (manual)	TC (manual)	UENS	Total mTCNS sensory score
IENFD distal leg	1.00								
IENFD proximal thigh	0.18	1.00							
IENFD distal leg to proximal thigh ratio	0.90^1^	−0.11	1.00						
CNFD (manual)	−0.17	0.21	−0.28	1.00					
CNBD (manual)	0.04	0.34	−0.15	0.74^1^	1.00				
CNFL (manual)	−0.03	0.31	−0.20	0.85^1^	0.91^1^	1.00			
TC (manual)	−0.04	−0.16	0.10	−0.16	0.04	−0.02	1.00		
UENS	−0.52^1^	−0.21	−0.37^1^	−0.11	−0.16	−0.19	0.20	1.00	
Total mTCNS sensory score	−0.49^1^	−0.14	−0.39^1^	0.06	0.07	−0.02	0.03	0.79^1^	1.00

SFN (*n* = 20)	IENFD distal leg	IENFD proximal thigh	IENFD distal leg to proximal thigh ratio	CNFD (manual)	CNBD (manual)	CNFL (manual)	TC (manual)	UENS: pinprick total	mTCNS: pinprick + temperature score
IENFD distal leg	1.00								
IENFD Proximal thigh	−0.05	1.00							
IENFD distal leg to proximal thigh ratio	0.68^1^	−0.45^1^	1.00						
CNFD (manual)	−0.09	0.07	−0.10	1.00					
CNBD (manual)	0.31	0.01	0.24	0.74^1^	1.00				
CNFL (manual)	0.17	0.06	0.13	0.80^1^	0.92^1^	1.00			
TC (manual)	0.02	−0.37	0.48^1^	−0.11	0.29	0.21	1.00		
UENS: pinprick total	−0.62^1^	−0.09	−0.22	0.20	−0.07	0.08	0.09	1.00	
mTCNS: pinprick + temperature score	−0.50	0.01	−0.51^1^	0.19	−0.02	0.05	−0.20	0.67^1^	1.00

CNFD, corneal nerve fiber density; CNBD, corneal nerve branch density; CNFL, corneal nerve fiber length; IENFD, intraepidermal nerve fiber density; mTCNS, modified Toronto Clinical Neuropathy Score; TC, tortuosity coefficient; UENS, Utah Early Neuropathy Screening.

^1^ Indicates statistical significance (*P* < 0.05) based on a two‐sided *P*‐value.

### Correlation among SFN measurements

Among participants with SFN (*n* = 20), there were significant positive correlations between manual CCM parameters (CNFD vs. CNBD: *r* = 0.74, CNFD vs. CNFL: *r* = 0.80, CNBD vs. CNFL: *r* = 0.92; Table 3). TC was positively correlated with the IENFD leg/thigh ratio (*r* = −0.48). Other manual CCM parameters did not correlate with IENFD or other clinical SFN scales (all *P* > 0.05). IENFD of the distal leg significantly and negatively correlated with UENS pinprick score (*r* = −0.62), but not with IENFD of the proximal thigh, mTCNS pinprick/temperature score. IENFD leg/thigh ratio was significantly and negatively correlated with mTCNS pinprick/temperature score (*r* = −0.51) but not UENS pinprick score.

### Participant preference

Of participants that completed CCM and skin biopsies (*n* = 129), 66 (52.0%) preferred skin biopsy and 61 (48.0%) preferred CCM. There were two participants who did not respond.

## Discussion

In participants with obesity, we found that IENFD of the distal leg had significantly higher diagnostic capability for DSP and SFN versus IENFD of the proximal thigh and 11 manual/automated CCM parameters. The superior performance as a DSP diagnostic of IENFD of the distal leg versus proximal thigh is expected based on the distal‐to‐proximal progression of neuropathy. Moreover, AUC of IENFD of distal leg for SFN was higher than for DSP, as might be anticipated for a metric that specifically assesses small fiber density. In addition, a simple screening questionnaire (MNSIq) had comparable diagnostic capability to IENFD of the distal leg and significantly better diagnostic capability for DSP and SFN compared to 11 CCM parameters and IENFD of the proximal thigh. Furthermore, among persons with DSP or SFN, IENFD of the distal leg had stronger correlations to clinical DSP/SFN scales compared to CCM parameters. Finally, patient preference was mixed, with just over half preferring skin biopsy to CCM.

To our knowledge, five previous studies directly compared the diagnostic capability of IENFD and CCM. A Danish study of 374 persons with and without Type 2 diabetes, found IENFD of the distal leg (AUC: 0.71) had better diagnostic capability for DSP by Toronto consensus definition versus CCM parameters (AUC: 0.52–0.55).^14^ A second Danish study of 680 adults with suspected neuropathy also found that IENFD of the distal leg (AUC: 0.74) had superior diagnostic characteristics compared to CCM (AUC: 0.63) measures for SFN or mixed fiber neuropathy as defined by Itani et al.^16^, ^29^ In addition, a US study of 168 patients with and without Type 2 diabetes found that IENFD (AUC: 0.74) had slightly larger AUC versus CCM measures (AUC: 0.67–0.68) for DSP.^15^ Although AUC were not calculated, a study of German adults with (*n* = 86) and without Type 2 diabetes (*n* = 48) also found low levels of correlation between IENFD and CCM parameters (Pearsons correlation coefficient range: 0.03–0.26).^30^ In contrast, two smaller studies of adults from the United Kingdom (*n* = 88 and *n* = 89) with and without Type 1 diabetes found that CCM parameters (AUC: 0.59–0.82) typically had better diagnostic capability for DSP by Toronto consensus definition, compared to IENFD of the dorsum of the foot (AUC: 0.66–0.73).^12^, ^13^ In summary, four of six studies, including our own herein, with head‐to‐head comparisons, comprising 1362 participants, found IENFD had superior diagnostic capability than CCM. In contrast, only two of six studies, totaling just 177 participants, found that CCM had improved diagnostic capability versus IENFD. Therefore, current evidence indicates that IENFD of the distal leg is the superior diagnostic measure for both DSP and SFN than CCM and should be the predominant outcome in research studies.

Although CCM has gained popularity as a less‐invasive alternative to skin biopsy,^31^ we found mixed patient preference for skin biopsy and CCM, with just over half of participants preferring skin biopsy. These findings do not support the main proposed advantage of CCM, namely patient preference, and suggest that researchers should select the diagnostic tool with the better diagnostic characteristics, that is, IENFD of the distal leg. Even if IENFD and CCM had the same diagnostic characteristics, then patients should be able to choose the test they undergo since patient preference between skin biopsy and eye procedures are evenly distributed. However, we and others^14^, ^15^, ^16^ have shown that IENFD of the distal leg has a clear diagnostic advantage over CCM, making the decision between these two tests straightforward.

We found that a simple questionnaire, namely the MNSIq, had similar diagnostic characteristics to IENFD of the distal leg, and significantly better characteristics compared to IENFD of the proximal thigh and all CCM metrics. This finding has important clinical and research implications. As a simple questionnaire, the MNSIq can be administered rapidly to assess DSP/SFN at the point‐of‐care without required training, expertise, or equipment, and, therefore, incurs lower healthcare costs. Moreover, the patient can fill out the MNSIq quickly and without any discomfort. Importantly, the MNSIq could even be assessed remotely, which allows a practical assessment of DSP/SFN in large clinical studies. In contrast, IENFD and CCM are difficult to rapidly assess DSP/SFN at the point‐of‐care, and require training, expertise, and equipment, and, consequently, incur higher costs. Further, CCM and IENFD both are relatively invasive; IENFD requires a skin biopsy and CCM requires eye numbing drops and a lubricant. In sum, the MNSIq has similar diagnostic capability to IENFD and better diagnostic capability than CCM and can be assessed rapidly at point‐of‐care, non‐invasively, and economically. Thus, the MNSIq is likely a better diagnostic test for DSP and SFN than either IENFD or CCM. Future device‐based diagnostic tests should be compared against simple measures, such as the MNSIq, to ensure that we only adopt new measures that are superior to a simple questionnaire.

Our study limitations include a cross‐sectional design and a sample size that was not large enough to internally validate with resampling techniques, such as cross‐validation. On the other hand, to date, this is the third largest study to assess IENFD in a head‐to‐head comparison to CCM, and the first to do so in a population with obesity. However, it remains unknown whether our results are generalizable to populations without obesity that may have different normative IENFD or CCM distributions. In addition, we did not assess diagnostic characteristics of IENFD and CCM for isolated SFN.

In summary, we found IENFD of the distal leg had better diagnostic capability for both DSP and SFN compared to IENFD of the proximal thigh and 11 CCM metrics. In addition, patient preference was mixed between completing IENFD versus CCM. Thus, IENFD of the distal leg offers the best quantitative assessment of DSP/SFN in research studies. Importantly, we found a simple questionnaire, the MNSIq, had strong diagnostic characteristics for DSP/SFN, demonstrating its potential as an easy to perform diagnostic tool for DSP/SFN.

## Author Contributions

Dr. Reynolds was involved in the study design, interpretation of the statistical analysis, and drafted the manuscript. Ms. Koenig, Ms. Kwiatek, Mr. Henderson, and Dr. Herrmann were involved in the collection of the study data and provided critical revisions of the manuscript. Ms. Watanabe performed and interpreted the statistical analysis and provided critical revisions of the manuscript. Dr. Elafros and Dr. Stino was involved in interpretation of the statistical analysis and critical revisions of the manuscript. Dr. Feldman was involved in the interpretation of the statistical analysis and critical revisions of the manuscript. Dr. Callaghan was involved in the study design, interpretation of the statistical analysis, and critical revisions of the manuscript. All authors have given final approval of the version to be published and agree to be accountable for all aspects of the work.

## Conflict of Interest

Dr. Reynolds, Ms. Koenig, Ms. Watanabe, Ms. Kwiatek, Dr. Elafros, Dr. Stino, Mr. Henderson and Dr. Feldman report no disclosures. Dr. Herrmannn discloses consulting over the past 3 years including Regenacy, Applied Therapeutics, DTx Pharma, Passage Bio, Roche, Pfizer, Orthogonal Neurosciences, NMD Pharma, GLG, Guidepoint Global, RM Global, and Keyquest Health. Dr. Callaghan consults for DynaMed, receives research support and editorial support from the American Academy of Neurology, and performs medical legal consultations including consultations for the Vaccine Injury Compensation Program.

## Data Availability

The data that support the findings of this study are available from the corresponding author upon reasonable request.
